# Expression of stemness genes in primary breast cancer tissues: the role of SOX2 as a prognostic marker for detection of early recurrence

**DOI:** 10.18632/oncotarget.1936

**Published:** 2014-05-01

**Authors:** Mauro Finicelli, Giovanni Benedetti, Tiziana Squillaro, Barbara Pistilli, Andrea Marcellusi, Paola Mariani, Alfredo Santinelli, Luciano Latini, Umberto Galderisi, Antonio Giordano

**Affiliations:** ^1^ Human Health Foundation, Spoleto, Italy; ^2^ Department of Medical Oncology,Macerata Hospital, Macerata, Italy; ^3^ Department of Statistics, University of Rome, Rome, Italy; ^4^ Department of Pathology,MacerataHospital, Macerata, Italy; ^5^ Department of Pathology Università Politecnica delle Marche, Ancona, Italy; ^6^ Sbarro Institute for Cancer Research and Molecular Medicine, Temple University, Philadelphia, PA, USA; ^7^ Department of Experimental Medicine, Second University of Naples, Naples, Italy

**Keywords:** Breast Cancer, Gene expression, Recurrence, SOX2, Stemness genes

## Abstract

The events leading to breast cancer (BC) progression or recurrence are not completely understood and new prognostic markers aiming at identifying high risk-patients and to develop suitable therapy are highly demanded. Experimental evidences found in cancer cells a deregulated expression of some genes involved in governance of stem cell properties and demonstrated a relationship between stemness genes overexpression and poorly differentiated BC subtypes.

In the present study 140 primary invasive BC specimens were collected. The expression profiles of 13 genes belonging to the OCT3/SOX2/NANOG/KLF4 core circuitry by RT-PCR were analyzed and any correlation between their expression and the BC clinic-pathological features (CPfs) and prognosis was investigated.

In our cohort (117 samples), NANOG, GDF3 and SOX2 significantly correlated with grade 2, Nodes negative status and higher KI67 proliferation index, respectively (p=0.019, p=0.029, p= 0.035). According to multivariate analysis, SOX2 expression resulted independently associated with increased risk of recurrence (HR= 2,99; p= p=0,004) as well as Nodes status (HR=2,44; p=0,009) and T-size >1 (HR=1,77; p=0,035).

Our study provides further proof of the suitable use of stemness genes in BC management. Interestingly, a prognostic role of SOX2, which seems to be a suitable marker of early recurrence irrespective of other clinicopathological features.

## INTRODUCTION

Despite recent medical advances, breast cancer (BC) remains the mostcommon neoplasm and the leading cause of cancer death in women. Patients with metastatic disease at the diagnosis represent approximately 6-10% while a further 20-50% will develop metastatic disease despite adjuvant and primary treatments [1]. Since the events leading to BC progression or recurrence within a variable time interval are not completely understood, it is not possible to accurately predict recurrence/ the development of metastasis. Thus, new prognostic markers aimed at identifying high-risk patients and enabling oncologists to developtailored treatment strategies are urgently needed.

Beyond the histological subtypes, the availability of immunophenotypical characteristics, gene expression profiling, molecular classification and recent advances in DNA sequencing technologies have led to an in-depth understanding of intra-tumor heterogeneity. This has resulted in useful prognostic and predictive information as well as new awareness about the complexity of each breast tumor subtype composed of cancer cells with different phenotypes at varying frequencies, which may change as the tumor evolves [2;3;4].

Over the last few decades, some studies have gone beyond BC subclassification, proposing a number of hypotheses to explain tumor recurrence, ranging from clonal selection to angiogenic dormancy [5]. Recently, new insights have been provided by the “cancer stem cell (CSC) hypothesis”. According to this hypothesis, many tumors, including BC, are hierarchically organizedand driven by a small population of cancer cells that displays stem cell properties such as self-renewal and pluripotency[6;7]. These cells have been considered responsible for tumor initiation, maintenance and multilineage differentiation as well as associated with drug resistance, tumor recurrence and metastasis [8;9;10]. CSC or tumor-initiating stem cell (T-ISC)-enriched populations have been identified by discrete surface markers and by their ability to generate tumor spheres and xenograft tumors with high frequency [11; 12; 13; 14; 15; 16]. Particularly in primary breast cancers, it has been demonstrated that CD44+CD24neg/low ESA+ cells are able of initiating xenograft models compared to bulk tumor cells [17].

Aldehyde dehydrogenase 1 (ALDH1) activity also marks breast cancer cell senriched forstem cell properties. The CSC model does not imply that tumors are generated from transformed tissue stem cells. The target of transformation could be a tissue stem cell, a progenitor cell, or a differentiated cell that acquires self-renewal ability. On the basis of the correlation between induced pluripotency reprogramming and cancer, it has been speculated that CSCs may arise through a reprogramming-like mechanism. Indeed, current evidence indicates some specific pluripotency genes, such as OCT4, SOX2 and NANOG, expressed in specific human cancer types as putative regulators of embryonic stem cell (ESC) identity [18;19]. Many studies have reported the expression of stemness genes in primary tumor tissues, thereby suggesting the possible existence of a population of cancer cells within the tumor mass that show stem cell-like properties and are actively involved in sustaining tumor growth and dissemination [20;21].

According to these studies, the expression signature of the stemness state of primary tumors couldrepresent a specific and reproducible method for identifying patients who are most likely to suffer recurrence or develop metastases and may also represent a specific target to be addressed in new therapeutic approaches [22]. In previous studies, we retrospectively analyzed the expression profiles of a panel of 13 stemness genes, in endometriotic and neuroblastoma tissues. Our data suggested a role of some of these genes in the progression of malignancy of both pathologies [23; 24]. In the present study, we analyzed the expression profiles of the same panel of stemness genes belonging to the OCT3/SOX2/NANOG/KLF4 core circuitry and acting in regulating stem cell biologyin a representative sample of primary breast cancer tissue. We also investigated whether there was any correlation between expression of stemness genes and BC clinicopathologicalfeatures and evaluated their potential predictive role as biomarkers for disease recurrence

## RESULTS

### Expression profile of stemness gene in BC primary tumor samples

**Figure 1:**
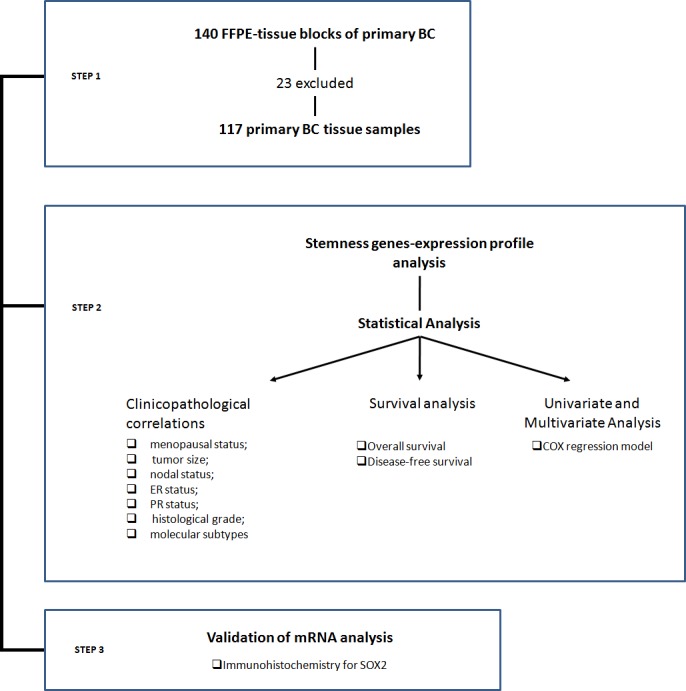
Workflow of the proposed research

**Figure 2:**
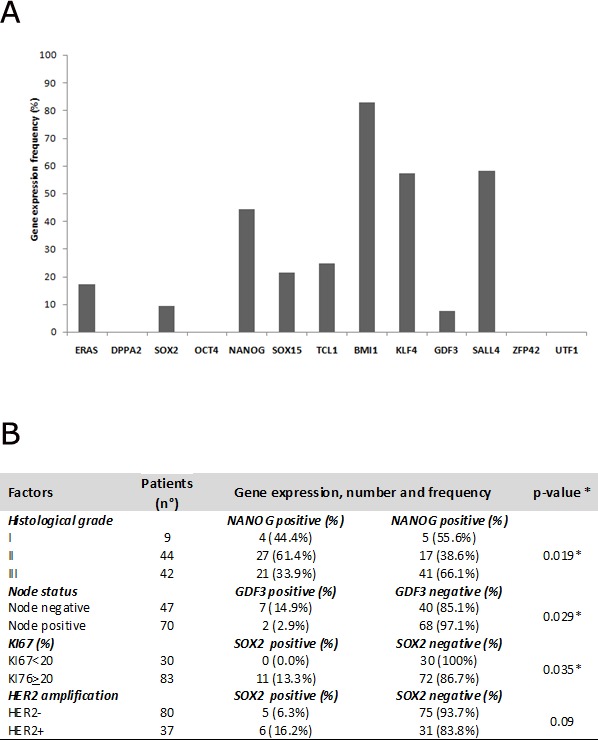
A) Histograms report gene frequency distributions calculated as the ratio between the number of patients expressing gene and the total number of the cohort (n=117); B) table reporting relationship between clinicopathological features and stemness genes * statistically significant result

The mRNA levels of the 13 genes, which are related to stemness properties, and of the two housekeeping genes, β-actin and PPIA, were detected by real-time reverse transcription PCR (RT-PCR) in 140 primary cancer tissues. According to amplification of β-actin and PPIA mRNA, 23 samples showing absent/low amplification were excluded from the analysis (Figure 1). The mRNA levels in the remaining 117 BC tissue samples are reported in Figure 2. Four stemness genes (DPPA, OCT4, ZFP42 and UTF1) were not detected in our analysis. Nine stemness genes were variably expressed as follows: GDF3 = 7.2% (9/117), SOX2= 9.4% (11/117), ERAS= 17.0% (20/117), Sox15 = 21.4% (25/117), TCL1= 24.8% (29/117), Nanog = 44.5% (52/117), KLF4 = 57.2% (67/117), SALL4 = 58.1 % (68/117), BMI1 = 83.0% (97/117) (Figure2A). It is worth noting that, to our knowledge, this is the first study reporting the expression of SOX15 and TCL1 mRNA in BC tissue.

### Correlations between pathological features and stemness gene expression

The relationship between mRNA expression profiles of the 9 stemness genes previously identified and the main clinicopathological features with prognostic significance was analyzed using the Chi-square test (or Fisher’s exact test when needed). Only statistically significant associations showing a p<0.05 were reported and summarized in Figure2B. In these analyses, NANOG mRNA expression was correlated with an intermediate tumor grade of invasiveness (G2), GDF3 mRNA expression with a node negative status and SOX2 mRNA expression with a higher KI67 proliferation index: all of them resulted statistically significant (p=0.019, p=0.029 and p= 0.035, respectively).

Interestingly, SOX2 expression seemed to be prevalent in HER2+ subtype tumors compared with HER2-: 6 out of 37 (16.2%) vs. 5 out of 80 (6.3%) (Figure2B), albeit not statistically significant (p =0.09).

Correlations between BC subtypes (luminal A, luminal B, HER2, triple negative) and stemness genes expression did not result statistically significant.

### Risk of recurrence and stemness gene expression

**Figure 3:**
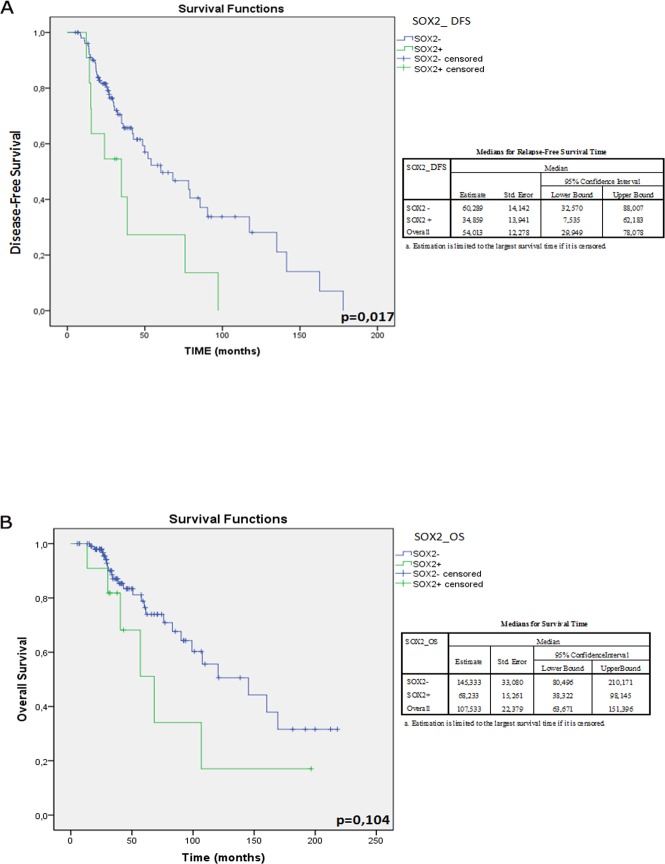
Kaplan-Meier plot representing the overall survival and disease-free survival of BC patients according to SOX2 mRNA expression profiles A) The disease-free survival rate of SOX2 + patients was significantly lower than that of SOX2-patients. B) In spite of a discernible trend of better survival of SOX2- patients this data is not statistically significant. On the left of the corresponding plot DFS- and OS-median values of the overall cohort and those stratified according to SOX2 amplification and those relative to the overall cohort were reported.

We used a univariate and multivariate Cox model for DFS and OS to identify the prognostic role of the expressed stemness genes. A statistically significant higher risk of recurrence was observed in 9 out of 11 patients (82%) with tumors expressing SOX2 mRNA (SOX2+) compared to 44 out of 106 (42%) without SOX2 expression (SOX2-) (p=0.017; χ^2^=5,740). Interestingly, SOX2+ patients experienced an earlier recurrence (median: 34.9 months; 95% CI: 7.5-62.2) than SOX2- patients (median: 60.3 months; 95% CI: 32.6-88.1), as shown by curves K-M in Figure 3A. Consistently, OS resulted shorter in SOX2+ compared to SOX2-: 145.3 months (95% CI: 80.5-210.2) vs. 68.2 months (95% CI: 63.7-151.4), respectively, albeit not statistically significant (p=0.104) (Figure 3B). The analysis of other genes (ERAS, NANOG, SOX 15, TCL1, BMI1, KLF4, SALL4 and GDF3) did not show any statistically significant correlation with DFS and OS (data not shown).

These data, along with the previous findings reporting the correlations between SOX2+ and higher KI67 index, support the possibility that SOX2 might play a promising role as a prognostic and predictive marker among the screened stemness genes.

**Figure 4:**
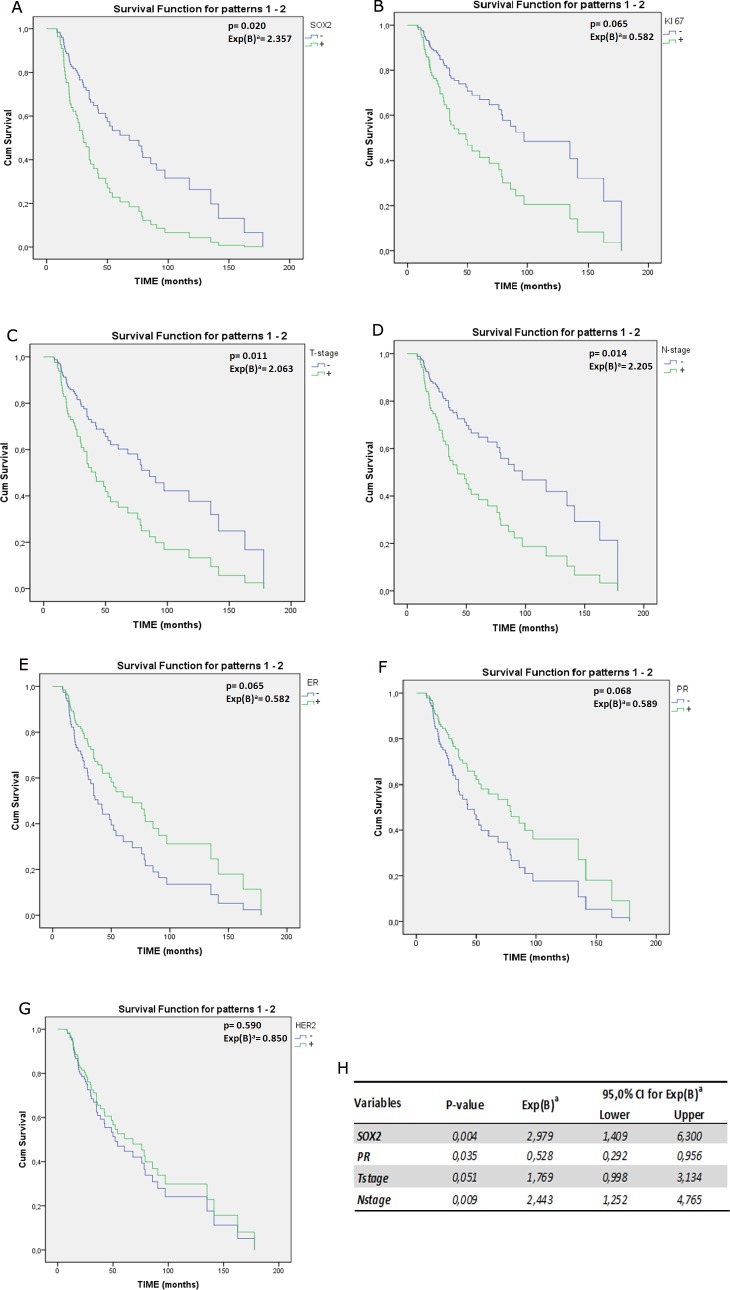
A-G) Survival plot representing the univariate analysis of DFS for SOX2 (A) and prognostic and predictive factors commonly used in BC clinical management: the proliferation marker KI67 (B), amplification of HER2, T-size and Node status (C-D), ER and PR (E-F) and HER2 amplification (G) p value and HR was reported in each plot; H) in table are summarized data obtained from multivariate analysis of DFS using the Cox regression model. Exp(B)^a^= Hazard ratio

With the goal of assessing the role of SOX2 as an independent prognostic factor of recurrence, we performed a univariate analysis of DFS for SOX2+ and the currently recognized prognostic and predictive factors of BC. SOX2+ (HR= 2.357; p= 0.0020); KI67+ (HR= 2.187; p=0.028); T-size+ (HR= 2.063; p= 2.011); Node-status+ (HR=2.205; p=0.014); ER+ and PR+ (HR=0.582 and HR=0.589, respectively) resulted statistically significant at univariate analysis (Figure 4A-G). Furthermore the same factors were included in a multivariate regression model to estimate the corresponding hazard ratio (HR) associated to each of them (Figure 4H). According to multivariate analysis, SOX2, T-size, N-stage and PR status were independently associated with risk of recurrence and, specifically, that risk increased by 3 times in SOX2+ (HR= 2.99; 95% CI 1.41-6.30; p=0.004) compared to SOX2- BCs (Figure 4H). Similarly, node metastases and a T-size >1 increased the risk of recurrence in our cohort (HR=2.44; 95%CI 1.25-4.76; p=0.009 and HR=1.77; 95% CI=0.99-3,13; p=0.051, respectively). Differently, PR expression appeared to be associated with a risk reduction (HR=0.57; 95% CI=0.53-0.29; p=0.035).

Interestingly, comparison between univariate and multivariate analysis shows a gain in HR values of SOX2+ (from 2.357 to 2.979, respectively). This is proof of the effect exerted by confounding factors in masking SOX2 effectiveness and, in turn, provides a further indication about its suitability as a higher independent predictor factor of relapse tendency. Overall, these data support the possibility that SOX2 could play a pivotal prognostic and predictive role in the poorest BC outcomes.

### Immunohistochemistry

Despite the significant body of literature describing predictive or prognostic mRNA profiles for cancer, some criticisms arise out of the lack of correlation between protein and transcription profiles. As such, we validated our RT-PCR data with IHC staining for Sox2 (Mehta S et al., TherAdv Med Oncol 2010).

To this end, IHC analysis was performed on FFPE samples belonging to the 11 BC tissues showing SOX2 mRNA-amplification and 20 tissues, randomly selected among the 104 BC samples not expressing SOX2 mRNA, in order to detect Sox2 protein expression.

Five consecutive cross sections for each tissue sample were analyzed and only cross sections with markedly brown-stained cells, showing a clear structure, were scored positive for Sox2 protein expression.

**Figure 5:**
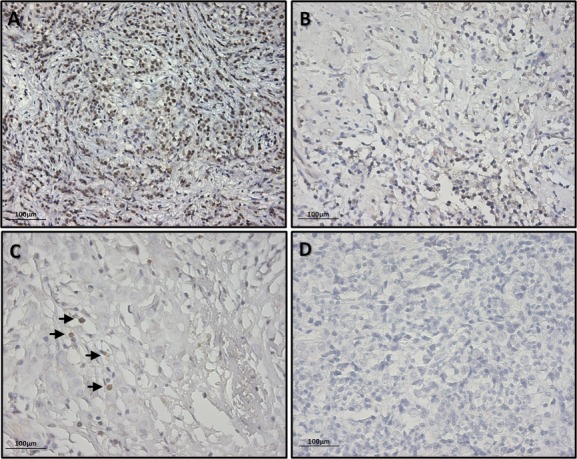
Exemplificative pictures of immunohistochemical staining of SOX2 showing different expression levels in BC tissues (A) Breast cancer tissue showing an high number of SOX2 positive cells; (B) Breast cancer tissue showing a moderate number SOX2 positive cells ; (C) Breast cancer tissue showing a low number SOX2 positive cells (black arrows); (D) Breast cancer tissue showing no SOX2 positive cells. Pictures were taken with 200X magnification.

IHC results showed a positive score for Sox2 protein expression, albeit with a different pattern of staining ranging from a high to a low number of positive cells (Figure 5A-C), in all of the 11 samples resulting in SOX2 mRNA amplification. Similarly, no Sox2-positive cells were found in the 20 samples randomly selected among the tissues not expressing SOX2 mRNA (Figure 5D).

Our data confirm a correlation between SOX2 mRNA and protein expression in our cohort of patients.

## DISCUSSION

Management of BC historically relies on the availability of well-established clinical and pathological factors and, recently, the gene expression profile has contributed togenerating prognostic and predictive data that could beuseful for choosing the most appropriate treatment.

The expression of genes regulating stem cell properties, such as self-renewal, pluripotency and uncommitted state, has been widely documented in cancer tissues (e.g., lung, brain, breast, prostate and colon), suggesting a possible prognostic role.

In previous studies, we reported that the analysis of the expression profile of genes involved in controlling stem cell properties in neuroblastoma and endometriosis samples provided interesting points for the development of new prognostic analyses [23; 24].

On the basis of the literature and our previous experience, we tested a panel of thirteen stemness genes in a significant number of BC tissue samples in order to understandhow specific TFs are expressed in BCs and whether they could significantly correlate with pathological characteristics and clinical outcome.

Three main results arose from our analysis. First, mRNA expression of SOX2 seems to be a prognostic factor for earlier relapse of BC. Second, mRNA expression of SOX2, NANOG and GDF3 correlates with specific pathological characteristics such as higher ki67 proliferation index, intermediate grade of invasiveness and absence of axillarylymph node metastasis. Finally, stemness genes are variably expressed in BC samples without any apparent correlation with pathological subtypes.

### SOX2 expression

The most relevant data in our analysis regard the prognostic role of SOX2 expression in BC patients. In particular, we demonstrated in our cohort that SOX2 expression correlated with a higher risk of tumor recurrence (p=0.017) and with a shorter disease-free survival compared to SOX2 tumors (median: 34.9 months; 95% CI: 7.5-62.2 vs. median: 60.3 months; 95% CI: 32.6-88.1, respectively). When the overall survival was considered, the Kaplan-Meyer curves showed a worse outcome in SOX2+ patients compared to SOX2- BC patients, but these data were not statistically significant. This maybe due to the fairly limited sample size, the fairly short follow-up and the different therapeutic choices beyond progression. To our knowledge, this is the first retrospective study reporting a direct correlation between mRNA amplification of SOX2 and PFS in BC patients.

Preclinical findings have shown the possible role of SOX2 in BC development and progression. Li et al. demonstrated that ectopical overexpression of SOX2 in MBA213 cell lines resulted in high infiltration potential by boosting the proliferation of metastatic cells on secondary tissue/organs of xenografted mice [29]. Indeed, SOX2 down-regulation in BC cell lines resulted in decreased tumor cell proliferation and colony formation [30].

Other studies have reported the amplification of the SOX2 gene in a variety of solid tumors with a possible role in cancer progression and prognosis [31; 32; 33; 34; 35]. In BCs, SOX2 expression has mainly been reported in basal-like subtypes, suggesting a role in conferring a less differentiated phenotype [36] and has been associated with potential tamoxifen resistance [37] A recent study by O Leis et al., which analyzed the expression of pluripotency genes (OCT4, NANOG and SOX2) by IHC in 158 BCs, demonstrated that Sox2+ tumors fell into the early stages (I-III) of tumor progression. Conversely, the expression of Oct4 or Nanog was not detected.

As a second step, we performed univariate analysis and multivariate analysis of DFS for SOX2 and six prognostic factors commonly used in clinical practice. SOX2 proved to be an independent prognostic factor and notably SOX2 expression increased the risk of recurrence by 3 times (HR= 2.98; 95% CI 1.40-6.30; p=0.004), irrespective of tumor size, nodal involvement and endocrine receptors. Interestingly, comparison between univariate and multivariate analysis shows a gain in the HR values of SOX2+ (from 2,357 to 2,979, respectively). This is proof of the effect exerted by confounding factors in masking SOX2 effectiveness and, in turn, provides a further indication about its suitability as a higher independent predictor factor of relapse tendency.

Multivariate analysis also showed an independent prognostic role of node metastases and a T-size >1 in increasing the risk of relapse, while PR expression exhibited a protective effect (HR=0.57; 95% CI= 0.53-0.29; p=0.035) (Figure 4H). These findings are consistent with data reported in the literature [38] and provide a further confirmation that our cohort was a representative sample of the heterogeneous BC population.

By evaluating correlations between clinicopathological features and SOX2 expression, we detected a significant association between mRNA amplification of SOX2 and high proliferation index in BC primary tumors (p=0.035) (Figure 2B). There is a growing body of evidence showing that high levels of Ki-67 are associated with worse prognoses and that Ki-67 positivity confers a higher risk of recurrence and a worse survival rate in patients with early breast cancer [39]. Therefore, our results on SOX2 expression were mutually consistent and identified a subpopulation of BC patients with a more aggressive disease and a poorer outcome irrespective of other prognostic factors.

Finally, it is worth considering that in this analysis SOX2 was one of the less expressed genes. mRNA amplification of SOX2 was found in a small group of our patients (9.4% of sample tissues) presenting heterogeneous clinicopathological characteristics and molecular subtypes. However, we observed a higher expression of SOX2 in the HER2+ subtype tumors compared to HER2-, 16.2% vs. 6.3%, respectively. Although not statistically significant and very preliminary, these data seem intriguing since recent studies have demonstrated that HER2 amplification increases the cancer stem cell population driving tumorigenesis and invasion, thus underpinning a role for HER2 in maintaining the cancer stem cell population [40; 41].

### NANOG and GDF3 expression

Unlike Leis and colleagues, who excluded NANOG expression by IHC in BC tissues, we detected mRNA amplification of NANOG in a large percentage of BC samples (44.4%). Moreover, in our study, the expression of NANOG resulted prevalent in tumors with an intermediate grade of invasiveness. Conversely, Ben-Porath and colleagues[19] found that activation targets of NANOG, OCT4, SOX2 and c-MYC are more frequently overexpressed in poorly differentiated tumors. There are at least two possible reasons for this discrepancy: first, in our cohort, the tumor grade was defined only on the basis of a morphological and partially reliable evaluation; second, according to the microarray-based analysis of BCs, G2 tumors have a hybrid signature, intermediate to G1 and G3 [42]. As a recent review highlighted, the expression of NANOG is higher in cancer stem cells than non-stemness cancer cells and its role in the stemness regulatory network is complex, as it is involved in the tumor initiation process, cross talks with several signal pathways, and in the communication between cancer cells and their surrounding stromal and immune cells [42].

We did not find a statistically significant correlation between NANOG expression and clinical outcome, unlike previous preclinical and clinical studies in BCs which reported a possible role of Nanog both as an indicator of a poor prognosis [44]and as a determinant of drug resistance in MCF-7 cells [45].

With regard to GDF3, in our cohort we found that its expression was limited to 7.7% of primary BC tumor samples and was associated with the absence of axillary lymph node metastasis (p=0.029), thus suggesting a protective effect of GDF3. Consistently, Li and colleagues showed that GDF3 proteins could inhibit the proliferation of MCF-7 and T47D cells and that the knockdown of GDF3 enabled colony formation and tumor progression in human BCs [46]. They further showed that over-expression of GDF3 in MCF7, a BC cell line stably expressing GDF3, could promote apoptosis induced by Taxolexposure. This evidence seems to agree with our data suggesting a correlation between GDF3 amplification and favorable BC outcomes.

### Molecular biology-based methods in diagnostics

It has been widely accepted that the diagnosis of cancer has undergone a paradigm shift, as cancer is no longer diagnosed based on morphological parameters alone. Advances in molecular biology technologies represent a valid alternative leading to the establishment of new routine methods and tools for investigating tumor biology and translating research findings into clinical practice [47]. The use of RT-PCR for marker detection in tumor tissues could help researchers and clinicians overcome some of the limitations associated with IHC-based procedures such as the high rate of false positives/negatives along with the lack of objectivity in interpreting results. Since 1988, when RNA extraction from FFPE tissues was first reported, many protocols have been described and standardization of extraction procedures has allowed for the use of these samples as a valuable resource for the analysis of RNA-based biomarkers [48].

Amplification of mRNAs belonging to the two housekeeping genes β-actin and PPIA was used to test RNA suitability [49], allowing us to eliminate23 samples from our analysis (Figure 1), and the short amplicon length of the PCR products (~ 150bp) guaranteed an efficient amplification (Supplementary file S1). These expedients allowed us to overcome biases due by the use of methods based on RNA extracted from FFPE tissue.

Moreover, to further confirm RT-PCR data relative to SOX2 amplification, IHC was performed on the same tissues and protein levels were evaluated. Our data revealed concordant results between the two techniques (Figure 5) and may represent further evidence confirming the suitability of a biology-based approach in cancer research and, in turn, provide new tools to improve/implement the current methodologies for cancer diagnosis.

## CONCLUSION

Our study provides further proof of the suitable use of stemness genes in BC management; however, there are certain limitations that should be taken into account when interpreting our results. First, the limited sample size must be considered. Second, with regard to pathological features, we relied on data collected at the time of diagnosis and did not perform a subsequent pathological review. Third, in the multivariate analysis of survival, we could not include the adjuvant treatments because of the number of variables already considered and the sample size. On the other hand, however, multivariate analysis revealed that our cohort was a representative sample of the heterogenous BC population.

In conclusion, we demonstrated: (i) a variable expression of stemness genes in heterogeneous BC samples; (ii) a statistically significant correlation of NANOG and GDF3 with pathological characteristics; (iii) a prognostic role of SOX2, which seems to be a suitable marker of early recurrence irrespective of other clinicopathological features.

## MATERIALS AND METHODS

### Patients and tumor characteristics

One hundred and forty BC tissue specimens were collected from 137 female patients who underwent diagnostic and curative surgery for invasive carcinomas from 1994 to 2011. Patients were selected from the institutional database of the Unit of Medical Oncology, Macerata Hospital (Italy). Institutional review board approval and expressed informed consent were obtained from all patients before sample collection. For the present study,specimes were harvested from the formalin-fixed paraffin-embedded sections of tumors. Upon diagnosis, BC tumor tissues were stained routinely with haematoxylin-eosin and reviewed by pathologists to determine the histological type according to WHO breast carcinoma histological classification criteria (2003), and clinical stage according to the UICC TNM classification (2003). Pathological stage was consideredfor patients who had undergone radical surgery, while clinical stage was taken into account in the other cases (locally advanced or metastatic disease). The cut-off for defining ER and PR positivity was established at 10% positivity for tumor cells observed by immunohistochemistry (IHC). Proliferation index was categorized as low if less than 20% of tumor cells stained positive for the nuclear antigen Ki67 and high if 20% or more were positively stained [25]. Human epidermal growth receptor 2- (HER2+) overexpression was defined as 2+ or 3+ using the DAKO HercepTest and confirmed by fluorescence in-situ hybridization (FISH) if 2+.

Tumors were considered as: basal-like (ER, PR and HER2-negative), luminal A (ER and/or PR-positive, HER2- negative and Ki67<20%), luminal B (ER and/or PR-positive, HER2-positive/negative, KI67≥20%) and HER2-positive subgroups.

After diagnosis, patients in the study received curative surgery, radiation therapy, adjuvant chemotherapy and hormonal treatment basing on histology, staging and risk of recurrence as foreseen using current guidelines. Patients were then managed as expected for standard follow-up procedures. Pathological and clinical characteristics are listed in Table 1. At a median follow-up of 41 months, 60 patients (43,8%) recurred. Median time to recurrence was 28 months (range 7to 177) and median survival from recurrence was 16,5 months (range 3 to 132).

**Table 1:** Patient and disease characteristics

Characteristic	Number of patients (N=137)	%
Median age	53 (range, 28 to 86)	
Hystotipe: -INVASIV DUCTAL -INVASIV LOBULAR -OTHERS	125 8 4	91.2 % 5.8 % 3.0 %
Grading: -G1 -G2 -G3 -UK	8 53 72 4	5.8 % 38.7 % 52.5 % 3.0 %
Endocrine receptors: -ER+/PR+ -ER+/PR− -ER−/PR+ -ER−/PR− -UK	61 33 3 39 1	44.5 % 24.1 % 2.1 % 28.5 % 0.8 %
HER2+ (IHC 3+ or FISH ampl)	38	27.7 %
KI67 amplification -KI67 ≣20 -KI67 <20 -UK	36 96 5	26.3 % 70.1 % 3.6 %
TNM at diagnosis: -T1 -T2 -T3 -T4 -TX -UK	64 45 13 11 3 1	46.7 % 32.8 % 9.5 % 8.1 % 2.1 % 0.8 %
-N neg -N pos -Nx	46 85 6	33.6 % 62.0 % 4.4 %
-M0 -M1	132 5	96.4 % 3.6 %
Surgery: -quadrantectomy -mastectomy -local excision	62 66 9	45.3 % 48.2 % 6.5 %
Radiotherapy	83	60.6 %
Adjuvant/neoadjuvant chemotherapy -CMF -ANTHRACYCLINES -TAXANES -ANTHRA+TAXANES -others	16 31 6 57 3	11.7 % 22.6 % 4.4 % 41.6 % 2.2 %
Adjuvant/neoadjuvant trastuzumab (HER2+)	27	19.7 %
Adjuvant endocrine therapy -TAM+/−LHRH analogs -AIs	37 59	27.0 % 43.0 %

### RNA extraction and RT-PCR analysis

Total RNA was extracted from four 10-μm sections from formalin-fixed, paraffin-embedded (FFPE) BC primary tissues with RNeasyFFPEkit (Qiagen Italia, Milano, Italy), according to the manufacturer’s instructions. A DNase I treatment step was included. RNA concentration was measured using a NanoDrop ND-2000 spectrophotometer (NanoDrop Technologies). Absence of residual genomic DNA was verified by polymerase chain reaction (PCR) on total RNA without reverse transcription (RT). Genomic human DNA was used as a positive control of PCR reactions.

cDNA was generated from 600 ng of each RNA sample. RT was done at 42°C for 1 h in the presence of random hexamers (Roche, Milan, Italy) and Avian Myeloblastosis Virus Reverse Transcriptase (Promega, USA). GeneBank sequences for human mRNAs *SOX2*, *SOX15*, *ERAS*, *SALL4*, *OCT4*, *NANOG*, *UTF1*, *DPPA2*, *BMI1*, *GDF3*, *ZFP42*, *KLF4*, *TCL1* and Primer Express software (Applied Biosystems, Foster City, CA, USA) were used to design primer pairs for the genes and the house keeping gene β*–actin* and *peptidylprolylisomerase A (PPIA)*. Primer sequences are listed in Supplementary file S1. PCR amplification was performed by using CFX96 real-time PCR (Bio-Rad,Hercules, CA, USA). Reactions were performed according to the manufacturer’s instructions using SsoFast™EvaGreen®Supermix (Bio-Rad,Hercules, CA, USA); melting curves (65°C–94°C) were generated to determine whether there were anyspurious amplification products. The real-time PCR efficiency was calculated for each primer pair using a dilution series and Bio-Rad analysis software. Appropriate regions of β*–actin* and *PPIA* cDNA were used as qualitative transcript controls. Each real-time PCR reaction was repeated at least three times and mRNA expression profiles were determined according to the ΔΔC_T_ method for relative quantitation (BioRad Software; Bio-Rad,Hercules, CA, USA) as long as the PCR efficiencies between the target mRNA and housekeeping mRNA wererelatively equivalent and close to 100%.

### Immunohistochemistry

Immunohistochemistry was performed to examine Sox2 expression in BC tissues. Briefly, 5μm thick sections were obtained with a microtome and transferred into adhesive slides. After deparaffinization and rehydration, sections were pretreated in 10mM sodium citrate buffer (pH 6.0) for antigen retrieval in a microwave oven for 20 minutes. After 1 h incubation in blocking solution (2% bovine serum albumin and 1% rabbit serum), slides were incubated overnight at 4°C with Sox2 mouse monoclonal antibody (1:50, Y17, Santa Cruz Biotechnology, USA). Sections were then incubated with 3% hydrogenous peroxide solution for 10 min to block endogenous peroxidase. Immunodetection was performed with biotinylated anti goat immunoglobuline (Santa Cruz) followed by peroxidase-labeled streptavidin (VectorLaboratories, Burlingame, CA, USA). Revelation of antibodies was performed by incubation with diaminobenzidine and HRP substratebuffer (Vector). Sections were counterstained with Mayer’s hematohylin (Sigma-Aldrich, St. Louis, MO, USA). To support the validity of staining, a negative control, in which the tissue was incubated with antibody diluents without the primary antibody included, was used for each reaction. Additional positive and negative tissue type controls, consisting in staining of tissue samples that are known to express or not express the epitope of interest, respectively, were used to support the species-specificity of the Sox2 antibody. To this end, fetal brain tissue and rat carotid sections were used as positive and negative tissue type controls, respectively [26; 27](Supplementary file S2).

Image screening and photography of serial cross sections were performed using a Leica IM 1000 System (Leica Microsystems,Wetzlar, Germany). Two blinded independent observersanalyzed the slides; only nuclear staining with clear borders was interpreted as a true positive: faint cytoplasmic staining, if present, was deemed negative.

### Statistical analysis

Statistical analysis was performed to define the association between the expression profile of the embryonic stemness genes and the following clinical, pathological and biological variables: histotype, grading, estrogen and progesterone receptor [ER, PR]; proliferating index evaluated by Ki67 staining, HER2 overexpression, breast cancer subtype, tumor size, node status, adjuvant and neoadjuvant chemotherapy, adjuvant hormonal therapy.

Fisher’s exact test and the χ2 test were used to assess the significance of the cross-tabulated data. Survival analysis were calculated with Kaplan-Meier life table curves, the log-rank (Mantel-Cox) test was used to compare disease-free survival (DFS) and overall survival (OS) over patients group stratified according to gene expression profiles. DFS was calculated from the date of diagnosis to the date of first recurrence (local, regional, distant, secondary breast or any other cancer, or death) or, for event-free patients, to the date of the last follow-up.

OS was calculated from first diagnosis to death or to the last follow-up. Cinical and pathological variables, unless already cathegorical, were dichotomized as follow: ER+ vs ER-, PR+ vs PR-, Ki67+ (IHC staining level > 20%) vs Ki67- (IHC staining level ≤ 20%), HER2+ vs HER2-, T size >1 cm vs T ≤ 1 cm; N- (no metastatic axillary lymphnodes) vs N+ (≥1 metastatic axillary lymphnodes). Univariate analysis of DFS for stemness genes and the currently recognized prognostic factors in BCs (ER, PR, Ki67, HER2, tumor size and metastatic axillary nodes) was performed. In order to identify the independent predictive factors, after having verified the proportional hazards assumption, the Cox regression model was utilized to assess the effects of each confounding variables such as the menopausal status, tumor size, nodal status, ER and PR status, histological grade, molecular subtypes and HER2 overexpression. Presence versus absence of dichotomous variable was considered as comparator in the regression model and Wald test was used to test the statistical significance difference. In the multivariate analysis, according to backward-stepwise model criteria, it was considered only the variables with a P < 0.10. Data Management and descriptive statistics were performed with GraphPad Prism, version 5.01 while Cox Regression analyses were performed with SPSS-Windows, version 18.

For all statistical tests, a two-tailed P-value < 0.05 was considered as statistically significant.

## SUPPLEMENTARY FIGURE AND TABLE


