# Supplementary material for: Expression of stemness genes in primary breast cancer tissues: the role of SOX2 as a prognostic marker for detection of early recurrence
Source: Oncotarget. 2014 May 1;5(20):9678–88. doi: 10.18632/oncotarget.1936 (PMC4259429; doi:10.18632/oncotarget.1936)
Supplement: Supplementary file 1 [file oncotarget-05-9678-s001.pdf]

Expression of stemness genes in primary breast cancer tissues: the role of SOX2 as a prognostic marker for detection of early recurrence

Supplementary Material

Gene	Primer position	Primer sequence	Annealing temperature (°C)	PCR product lenght (bp)
**SOX2** *SRY (sex determining region Y)-box 2*	1563	5′-CCATCCACACTCACGCAAAA-3′	59	139
	1701	5′-TATACAAGGTCCATTCCCCCG-3′		
**OCT4** *POU class 5 homeobox 1*	1121	5′-TCCCATGCATTCAAACTGAGG-3′	60	103
	1223	5′-CCAAAAACCCTGGCACAAACT-3′		
**NANOG** *Nanog homeobox*	1169	5′-TGGACACTGGCTGAATCCTTC-3′	59	142
	1310	5′-CGTTGATTAGGCTCCAACCAT-3′		
**KLF4** *Kruppel-like factor 4 (gut)*	1508	5′-CTGCGGCAAAACCTACACAA-3′	60	182
	1689	5′-GGTCGCATTTTTGGCACTG-3′		
**ERAS** *ES cell expressed Ras*	969	5′-AATGTAGACCTTTCCCCAGGC-3′	58	135
	1103	5′-AAAGCCCCTCACCAAGTGAA-3′		
**GDF3** *Growth differentiation factor 3*	778	5′-AAAAGGAAGAGCAGCCATCCCT-3′	60	110
	887	5′-GCAATGATCCACTTGTGCCAA-3′		
**SOX15** *SRY (sex determining region Y)-box 15*	315	5′-GAACAGGTTGGAAGCAAAGGC–3′	59	127
	441	5′-GCGTCGATCCTGAAAATGGA-3′		
**DPPA2** *Developmental pluripotency associated 2*	798	5′-AGCCATGTTGGCATCATGG-3′	58	108
	9025	5′-GAGGCTTGCAGCAAAAAGGC-3′		
**SALL4** *Sal-like 4 (Drosophila)*	2394	5′-GCCCAGATATCCTGGAAACCA–3′	60	115
	2509	5′-TTCTCGGAGCTCTCTGCTTTG-3′		
**TCL1** *T-cell leukemia/lymphoma 1A*	667	5′-CTCGGCTTTTTCTCAGCTGGAT-3′	59	127
	793	5′-GGTGAATCGGCTGTGTTCTCA-3′		
**ZFP42** *ZFP42 zinc finger protein*	953	5′-ATGACAGTCTGAGCGCAATCG-3′	60	133
	1085	5′-AACGCTTTCCCACATTCCG-3′		
**UTF1** *Undifferentiated embryonic cell transcription factor 1*	876	5′-CGACATCGCGAACATCCTG-3′	64	117
	992	5′-AGAATGAAGCCCACGGCCA-3′		
**BMI1** *BMI1 polycomb ring finger oncogene*	437	5′-AATGTCTTTTCCGCCCGCT-3′	59	139
	575	5′-ACCCTCCACAAAGCACACACAT-3′		
**ACTBL2** *Actin, beta-like 2*	926	5′-CCATCATGAAGTGTGACGTGG-3′	61	102
	1027	5′-TCTGCATCCTGTCGGCAAT-3′		
**PPIA** *Peptidylprolyl isomerase A (cyclophilin A)*	417	5′ – CAAATGCTGGACCCAACACA – 3′	*58*	*72*
	488	5′ – TGCCATCCAACCACTCAGTCT – 3′		

Supplementary file S1: Primers for real time RT-PCR

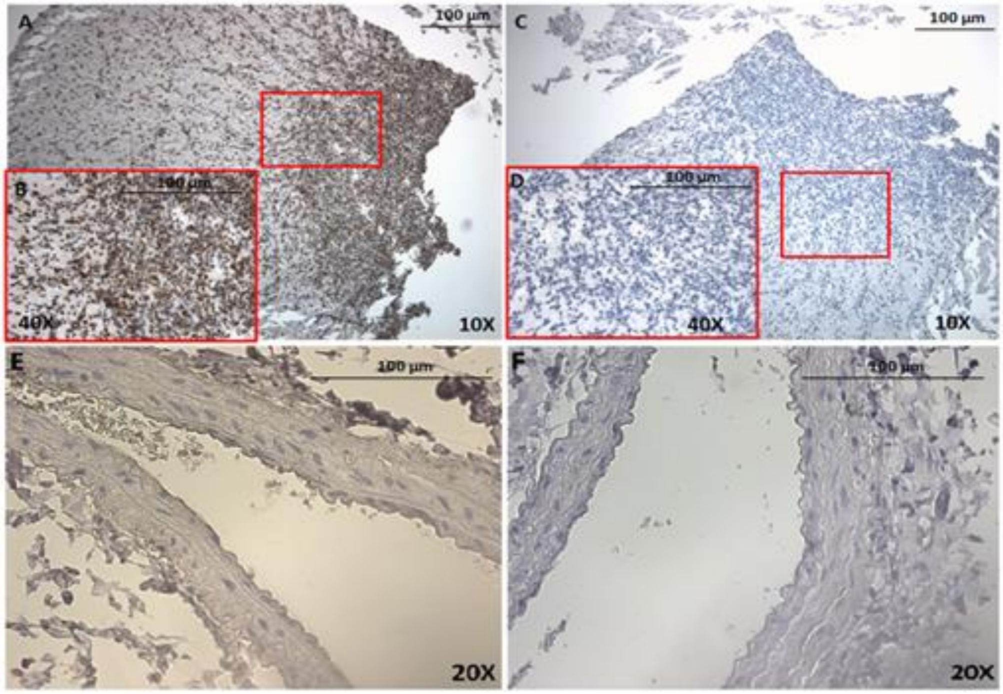

Supplementary figure S2: Immunohistochemical staining performed to support the species-specificity of SOX2 antibody. Representative pictures of SOX2 immunohistochemistry in human fetal brain (A-B)and in rat carotid (E) chosen as positive and negative tissue type controls, respectively. To support the validity of staining a negative control, in which the tissue was incubated with antibody diluents without the primary antibody included, was performed for both fetal brain (C-D) and rat carotid (F).
